# *Klebsiella pneumoniae*: an increasing threat to public health

**DOI:** 10.1186/s12941-019-0343-8

**Published:** 2020-01-09

**Authors:** Clement Yaw Effah, Tongwen Sun, Shaohua Liu, Yongjun Wu

**Affiliations:** 1 College of Public Health, Zhengzhou University, Zhengzhou, 450001 China; 2 General ICU, The First Affiliated Hospital of Zhengzhou University, Henan Key Laboratory of Critical Care Medicine, Zhengzhou, 450052 China

**Keywords:** *Klebsiella pneumoniae*, Antibiotic resistance profiles, Resistant genes, Virulent genes, Risk factors, Asia

## Abstract

**Objectives:**

This review fills the paucity of information on *K. pneumoniae* as a nosocomial pathogen by providing pooled data on epidemiological risk factors, resistant trends and profiles and resistant and virulent genes of this organism in Asia.

**Methods:**

Exhaustive search was conducted using PubMed, Web of Science, and Google scholar for most studies addressing the prevalence, risk factors, drug resistant-mediated genes and/or virulent factors of *K. pneumoniae* in Asia. Data extracted for meta-analysis were analyzed using comprehensive meta-analysis version 3. Trends data for the isolation rate and resistance rates were entered into Excel spread sheet and the results were presented in graphs.

**Results:**

The prevalence rate of drug resistance in *K. pneumoniae* were; amikacin (40.8%) [95% CI 31.9–50.4], aztreonam (73.3%) [95% CI 59.9–83.4], ceftazidime (75.7%) [95% CI 65.4–83.6], ciprofloxacin (59.8%) [95% CI 48.6–70.1], colistin (2.9%) [95% CI 1.8–4.4], cefotaxime (79.2%) [95% CI 68.0–87.2], cefepime (72.6) [95% CI 57.7–83.8] and imipenem (65.6%) [95% CI 30.8–89.0]. TEM (39.5%) [95% CI 15.4–70.1], SHV-11 (41.8%) [95% CI 16.2–72.6] and KPC-2 (14.6%) [95% CI 6.0–31.4] were some of the resistance mediated genes observed in this study. The most virulent factors utilized by *K. pneumoniae* are; hypermucoviscous phenotype and mucoviscosity-related genes, genes for biosynthesis of lipopolysaccharide, iron uptake and transport genes and finally, adhesive genes.

**Conclusion:**

It can be concluded that, antimicrobial resistant in *K. pneumoniae is* a clear and present danger in Asia which needs strong surveillance to curb this menace. It is very important for public healthcare departments to monitor and report changes in antimicrobial-resistant isolates.

## Background

The problem of antibiotic resistance has become an albatross on the neck of clinicians, veterinarians and other infection control agents in their quest to treat and prevent infections caused by microorganisms that were once thought to have been eradicated with antimicrobials. These organisms or superbugs are returning in new forms resistant to almost all clinically important antimicrobials. Unfortunately, the pharmaceutical pipeline merely does not have enough new medicines to maintain pace with drug-resistant bacterial infections [1]. *Klebsiella pneumoniae* is one of such clinically significant organisms that have acquired much public health concern. *Klebsiella pneumoniae* is a significant *Enterobacteriaceae* considered as one of the opportunistic pathogens causing broad spectra of diseases and showing increasingly frequent acquisition of resistance to antibiotics.

According to Shiri et al. [2], this organism accounts for about one-third of all Gram-negative infections such as urinary tract infections, cystitis, pneumonia, surgical wound infections, endocarditis and septicemia. It also causes necrotizing pneumonia, pyogenic liver abscesses and endogenous endophthalmitis [3]. High mortality rates, extended hospitalization, coupled with high cost are often associated with infections caused by this organism [4]. The drastic rise in the incidence of multidrug-resistant (MDR) and extremely drug-resistant (XDR) pathogens belonging to the *Enterobacteriaceae* group is a major economic problem as these pathogens are prevalent natural residents of human and animal microbiome. Despites its numerous clinical importance, there is still paucity of information on *K. pneumoniae.*

This review was therefore designed to determine the antibiotic-resistant profiles of *Klebsiella pneumonia* as a nosocomial pathogen and focuses on some differences between classical and non-classical subtypes, antimicrobial resistance-mediated genes, some virulent factors of this organism, and some epidemiological risk factors through a systematic review and meta-analysis. This review also looked at some trends in the isolation and resistance rates of *K. pneumoniae* using China as the target country.

## Methods

### Search strategies

Exhaustive search was conducted using PubMed, Web of Science, and Google scholar for most studies addressing the prevalence and/or the molecular epidemiology of drug resistant strains of *K. pneumoniae* in some selected countries in Asia. The search filtered articles among the years of 2005 to 2019. The applied keywords included *Klebsiella pneumoniae,* antibiotic resistance, resistant genes, virulent genes, epidemiological risk factors and Asia.

### Inclusion and exclusion criteria

The original published articles on the prevalence of drug resistant strains of *K. pneumoniae* from hospital-acquired infections in some selected countries in Asia were considered. Before an article will be considered useful to this study, its antibiotic susceptibility testing should use reference standard methods and recommendations by the Clinical and Laboratory Standards Institute (CLSI) for drug susceptibility testing of *K. pneumoniae* against most commonly used antimicrobial agents. Due to the following reasons, some studies were excluded from this studies; articles not following CLSI recommended drug susceptibility testing methods, case reports, meta analyses or systematic reviews, letters to editor, review articles, non-English, and duplicate publication.

### Data extraction

For the meta-analysis, information extracted from each article were authors’ name, the publication time, year of study, number of samples, antimicrobial drug resistant profiles of *K. pneumoniae*, resistance-mediated genes, and virulent genes.

### Statistical analysis

The comprehensive Meta-analysis software version 3 was used to analyze the data. Because of the heterogeneity among studies, random effects models was used and tested with the Cochrane Q test. For trends in antimicrobial resistance and isolation rates, data were entered into excel spread sheet and graphs were plotted based on the available data.

## Results

**Fig. 1:**
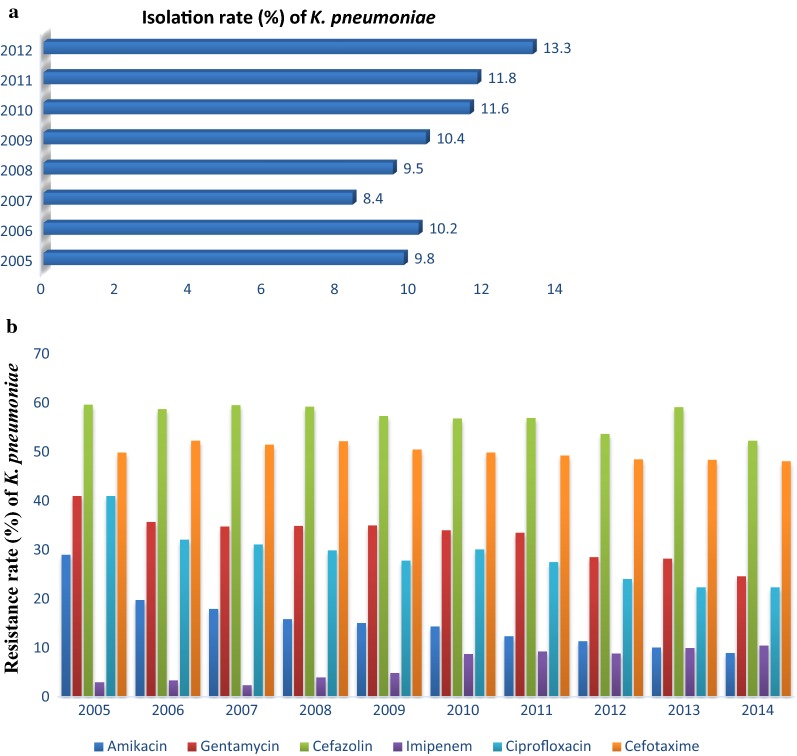
Trends of resistance and isolation rate of *K. pneumoniae* in China. **a** The isolation rate of *K. pneumoniae* in China (Data extracted from CHINET surveillance system 2015 which collected data from outpatients and inpatients in 19 big hospitals from 14 provinces. **b** The resistance rate of *K. pneumoniae* commonly used antimicrobials in China (Data extracted from CHINET surveillance system 2015 which collected data from outpatients and inpatients in 19 big hospitals from 14 provinces for a period of 10-year [57]

From the literature search, a total of 143 studies or articles were screened from PubMed, Web of Science, and Google scholar. After the removal of duplicates and evaluation of titles and abstract, 64 full text scripts were evaluated. After secondary full text evaluation, 20 studies or articles (Table 1) addressing the prevalence, drug resistant-mediated genes and/or virulent factors in *K. pneumoniae* in Asia were selected for the final meta-analysis. From Fig. 1, it can be seen that there is an increasing trend in the isolation rate of *K. pneumoniae (*from 9.8% in 2005 to 13.3% in 2012) in China. Interestingly, the isolation rate decreased in 2007 but increased in the preceding years. In contrast, the resistant trends of *K. pneumoniae* in China was not congruent to the isolation rate as there were decreasing resistance trends from 2005 to 2014. Imipenem recorded the lowest resistance rate but its resistance trends tend to increase steadily from 2005 to 2014. From Table 2, it can be seen that *K. pneumoniae* has a great resistance rate to most of the commonly used antimicrobials. Cefotaxime recorded the highest prevalence (79.2%) followed by aztreonam (73.3%) and cefepime (72.6%). Colistin recorded the lowest resistance rate of 2.9%. The individual studies as seen in Table 3, details the number of isolates which were resistant to these antimicrobials. These numbers in terms of percentage resistance to the various antimicrobials ranges from ‘’no isolate’’ (0%) to ‘’all isolates’’ (100%). From Table 4, it can be seen that *K. pneumoniae* harbor some genes that confers most of its resistance properties. In this review, the selected resistant-mediated genes were in the decreasing order of CTX-M-1 (41.9%), SHV-11 (41.8%), TEM (39.5%), CTX-M-15 (35.3%), KPC-2 (14.6%) and NDM-1 (6.7%). The genes, Mag, Armp, ArmpA2, allS (hypermucoviscous phenotype and mucoviscosity-related genes); wabG, uge, wcaG (biosynthesis of lipopolysaccharide genes); iutA, icuA, iroN, iroB, ybtA, irp2, kfu, entB (iron uptake and transport genes) and Cf29a, fimH, mrkD (Adhesion genes) are all some virulent factors that are used by *K. pneumoniae* to cause various harm or infections (Table 5).

**Table 1:** Details of articles included in the meta-analysis

Ref nos.	Refs	Publication year	Enrolment time	Province/country	No. of K.P isolates (N)	Type of K.P strain used (n)
[30]	Liu et al.	2019	2013–2017	Anhui/China	106	CRKP (106)
[39]	Tian et al.	2018	2016–2017	Shanghai/China	170	CRKP (170)
[40]	Zhao et al.	2019	2015–2016	Anhui/China	63	CRKP (63)
[41]	Meng et al.	2019	2014–2015	Central China	142	CRKP (142)
[42]	Kim et al.	2019	2016–2017	South Korea	579	KP (579)
[43]	Xu et al.	2019	2013–2015	Dalian/China	30	ESBL-P KP (30)
[44]	Guo et al.	2017	2009–2014	Henan/China	8203	KP (8203)
[29]	Dong et al.	2018	2011–2014	Beijing/China	146	CRKP (52)
[45]	Cha et al.	2018	2010–2014	Seoul/Korea	260	ESBL:AmpC-KP (54)
[46]	Alizade et al.	2018	2014–2015	Kerman/Iran	103	K.P (103)
[47]	Lu et al.	2018	2015–2016	Sichuan/China	112	HvCoR-KP (5)
[48]	van Dorp et al.	2019	2016–2017	Beijing/China	100	CRKp (100)
[49]	Shanker et al.	2018	2015	India		HvKP
[50]	Huang et al.	2018	2012–2014	Taipei/Taiwan China	63	HvCR-KP (63)
[51]	Abrar et al.	2019	2014–2017	Lahore/Pakistan	124	KP (124)
[52]	Mitra et al.	2019	2012–2014	Kolkata/India	55	KP (55)
[53]	Gautam et al.	2019	2014–2016	New Delhi, Chandigarh, Vellore, Puducherry/India	304	ESBL-KP and Non-ESBL-KP (304)
[54]	Mansury et al.	2016	2012–2013	Shiraz/Iran	38	ESBL KP (38)
[55]	Heidary et al.	2017	2013–2014	Tehran/Iran	117	K.P (117)
[56]	Ma et al.	2015	2012–2014	Taiwan China	760	CnSKP (760)

HvCoR-KP, hypervirulent colistin-resistant *Klebsiella pneumonia*; CRKP, carbapenem-resistant *Klebsiella pneumoniae*; hvKP, hypervirulent *Klebsiella pneumoniae*; HvCR-KP, hypervirulent carbapenem-resistant *Klebsiella pneumoniae*; CnSKP, carbapenem non-susceptible *Klebsiella pneumoniae*; K.P, *Klebsiella pneumoniae*; ESBL-P KP, extended spectrum β-lactamase producing *Klebsiella pneumoniae*

**Table 2:** Overall resistance rate of *Klebsiella pneumoniae* to various antimicrobials

Subgroups	No. of events/studies	Prevalence of drug resistance, % (*CI*)	Heterogeneity test	
			*I*^*2*^ (%)	*p* value
Overall resistant to amikacin	11	40.8 (31.9–50.4)	93.7	< 0.001
Overall resistant to aztreonam	9	73.3 (59.9–83.4)	97.4	< 0.001
Overall resistant to ceftazidime	10	75.7 (65.4–83.6)	90.5	< 0.001
Overall resistant to ciprpfloxacin	11	59.8 (48.6–70.1)	96.4	< 0.001
Overall resistant to colistin	5	2.9 (1.8–4.4)	0.0	0.5250
Overall resistant to cefotaxime	8	79.2 (68.0–87.2)	93.8	< 0.001
Overall resistant to cefepime	8	72.6 (57.7–83.8)	96.9	< 0.001
Overall resistant to gentamicin	9	58 (49.2–66.3)	89.7	< 0.001
Overall resistant to imipenem	10	65.6 (30.8–89.0)	99.6	< 0.001
Overall resistant to levofloxacin	6	54.1 (36.0–71.2)	92.7	< 0.001
Overall resistant to meropenem	11	62.7 (31.1–86.2)	99.5	< 0.001
Overall resistant to trimethoprim_sulfamethoxazole	7	58.2 (35.5–77.9)	98.8	< 0.001

CI, confidence interval; n, number of events (drug resistance); N, total number of *Klebsiella pneumoniae* from the included studies

**Table 3:** The prevalence of antimicrobial drug resistance among *Klebsiella pneumoniae* isolates according to individual studies

References	N	AMK	ATM	CAZ	CIP	COL	CTX	FEP	GEN	IPM	LVX	MEM	SXT
Liu et al. [30]	106	33	49	106	49	0	–	92	33	106	49	106	16
Tian et al. [39]	170	99	161	170	107	5	170	–	105	163	105	162	139
Zhao et al. [40]	63	48	61	62	59	–	–	58	50	62	56	63	–
Meng et al. [41]	142	45	89	78	67	–	–	81	69	–	60	–	42
Kim et al. [42]	579	–	139	–	141	–	–	–	–	–	–	–	131
Xu et al. [43]	30	16	–	29	26	–	28	24	–	–	23	–	–
Guo et al. [44]	8203	2568	4323	4077	4184	–	6005	2887	4651	468	–	476	5357
Dong et al. [29]	52	3	47	52	8	0	–	51	37	46	4	49	47
Cha et al. [45]	54	–	–	53	51	–	52	34	–	13	–	7	–
Mitra et al. [52]	55	45	50	–	52	–	53	–	52	–	–	29	49
Gautam et al. [53]	304	109	–	159	115	8	157	155	–	103	–	120	–
Mansury et al. [54]	38	6	–	–	–	–	19	–	16	6	–	4	–
Heidary et al. [55]	117	40	75	73	–	5	77	–	51	28	–	28	–
Ma et al. [56]	760	–	–	–	–	–	–	–	–	568	–	519	–
Sum		3012	4994	4859	4859	18	6561	3382	5064	1563	297	1563	5781
Rate (%)		40.8	73.3	75.7	59.8	2.9	79.2	72.6	58	65.6	54.1	62.7	58.2

AMK, amikacin; ATM, aztreonam; CAZ, ceftazidime; CIP, ciprofloxacin; COL, colistin; CTX, cefotaxime; FEP, cefepime; GEN, gentamicin; IPM, imipenem; LVX, levofloxacin; MEM, meropenem; SXT, trimethoprim-sulfamethoxazole

**Table 4:** The prevalence of some resistant-mediated genes among *Klebsiella pneumoniae* isolates

Subgroups	No. of events/studies	Prevalence of resistant gene % (*CI*)	n/N	Heterogeneity Test	
				*I*^*2*^ (%)	*P*-value
Overall prevalence of TEM genes	5	39.5 (15.4–70.1)	241/484	96.6	< 0.001
Overall prevalence of SHV-11 genes	7	41.8 (16.2–72.6)	350/1117	98.1	< 0.001
Overall prevalence of CTX-M-1 Genes	5	41.9 (21.6–65.4)	229/826	95.7	< 0.001
Overall prevalence of CTX-M-15 genes	5	35.3 (17.1–58.9)	153/548	95.0	< 0.001
Overall prevalence of KPC-2 genes	6	14.6 (6.0–31.4)	301/2031	97.1	< 0.001
Overall prevalence of NDM-1 genes	5	6.7 (1.7–23.4)	108/1452	95.6	< 0.001

CI, confidence interval; n, number of events (resistance-mediated genes); N, total number of *Klebsiella pneumoniae* from the included studies

**Table 5:** Some selected virulent factors encoded by different strains of *Klebsiella pneumoniae*

Virulence factors	References
Hypermucoviscous phenotype and mucoviscosity-related genes magA rmpA rmpA2 allS	[30, 42, 47–50]
Biosynthesis of lipopolysaccharide wabG uge wcaG	[42]
Iron uptake and transport iutA icuA iroN iroB ybtA irp2 kfu entB	[30, 42, 47–50]
Adhesion Cf29a fimH mrkD	[42, 47, 49, 50]

### *Klebsiella pneumoniae*: the classical and other subtypes

*Klebsiella pneumoniae* can be broadly classified into two subtypes; classical *Klebsiella pneumoniae* (cKp) and non-classical *Klebsiella pneumoniae* (ncKp). The antimicrobial resistance profiles and the virulence profiles of these strains vary with the former tagged as notorious [3, 5]. Notwithstanding, several clones of these ncKp have also been implicated in causing severe and difficult to threat infections due to their continuous mutation and the acquisition of plasmids and transposons which carries resistant and virulent genes. This has led to the emergences of strains such as hypervirulent *Klebsiella pneumoniae* (hvKp) or hypermucoviscous *Klebsiella pneumoniae* (HMKP). This strain was first identified in Eastern part of Asia and has since spread worldwide [6]. This subtype is non-resistant to most of the commonly used antimicrobials such as colistin and carbapenems. But the recent reports of carbapenem-resistant hvKp strains which belong to the sequence types 11 (ST11) [7], ST25 and ST65 [8] poses a major clinical concern.

HvKp strains can cause serious infections in both immunocompetent, diseased and healthy young individuals [9]. This hvKp is known to habour (i) sidephore; predominant of which is aerobactin which is concomitant with hypermucoviscosity, (ii) virulent factors such as; K1, K2, K20 capsular types, rmpA and rmpA2 mucoid-regulator genes [10]. The horizontal transfer of these plasmids and transposons has led to the multidrug resistance (MDR) and the extremely drug resistance (XDR) nature of most of these subtypes. The high prevalence rate of MDR and XDR *K. pneumoniae* subtypes reflects a multifactorial dissemination processes that include but not limited to: the spread of high risk global multi-resistant genetic lineage [11]; acquisition of successful multi-resistant plasmids; and acquisition of resistant genes located on successful transposons. Klebsiella is a major source of carbapenem resistance worldwide by the dissemination of its plasmids which is facilitated by high genetic transfer (HGT) to other species. Spread of these extended-spectrum β-lactamase (ESBL) and Carbapenemase-encoding plasmids poses a major threat, as acquisition of these plasmids turn bacteria into MDR and XDR. In China, the most dominant MDR KPC-producing clone is the ST11. Once the bla_KPC-2_ gene is introduces into a certain location, especially in a hospital setup, under antibiotic selection pressure, further dissemination of this gene may occur which may lead to MDR and XDR strains. Hypermucoviscous *K. pneumoniae* (HMKP) strain types which are sporadically distributed in Asia and the Middle East are the NDM-producing isolates [12, 13]. Liu et al. [14], had reported the first outbreak of CR-HMKP strains which harbored bla_NDM-1_ gene, shared the same pulsotypes (PT) and belong to the same Sequence types (ST).

### Epidemiological risk factors associated with *K. pneumoniae* colonization and infections

It is believed that several factors can cause the colonization of *K. pneumoniae* in a community as well as in a hospital setting. The cases of *K. pneumoniae* infections vary from country to country. In a study by Ling et al. [15], it was reported that, Chinese people had a colonization rate of 66.0% compared to Malay (14.3%), Indian (7.9%) and others nationals (11.8%). This is an indication that infections caused by *K. pneumoniae* can be locality specific although there can be some form of intercontinental similarities.

Some epidemiological risk factors associated with *K. pneumoniae* infections may include socio-demographic factors such as gender, age, hospitalization status, source of domestic water (river, rain, well, bottled, piped, boiled), companion animals (cats, dogs, birds), livestock (chicken, ducks, pigs, cow or water buffalo), malnutrition, co-morbidity and the use and misuse of detergents and antiseptics. In Asia, it has been reported that exposure to health care facility and history of previous overseas hospitalization (OR: 33.667; 95% CI 4.539–259.700) is one of the notifiable risk factors associated with *K. pneumoniae* colonization but this had been disagreed by Ling et al. [15], who iterated that persons with no history of overseas travel and overseas hospitalization are also at risk of *K. pneumoniae* colonization and infections, hence, suggesting that *K. pneumoniae* is a persistent organism in our community setting.

Also, admission to an ICU facility (OR: 11.899; 95% CI 4.986–28.399), antimicrobial exposure; particularly carbapenems and fluoroquinolones [14, 16], hematology patients and patients with immunodeficiency are all high risk factors for *K. pneumoniae* colonization and infections. Carrier levels in hospitalized patients are significantly higher, with reported rates of 77% in the stool, 19% in the pharynx, and 42% on the hands [3]. The higher rates of colonization are primarily related to the increasing use of antibiotics [3, 17, 18]. The increase in colonization rate of *K. pneumoniae* as observed in these clinical samples is of epidemiological important because, Klebsiella nosocomial infection was four times higher in stool carriers compared with non-carriers [19]. In a study in Taiwan, antibiotic use (e.g. ampicillin or amoxicillin) within the last 30 days was associated with an increased risk of liver abscess [20], suggesting that an increasing exposure to antimicrobials and the last period of antimicrobial administration is a major risk factor for *K. pneumoniae* colonization and infection.

In a study by Saleem et al. [21], it was reported that some risk factors associated with *K. pneumoniae* sepsis and mortality in a neonate intensive care units in Pakistan were; extremely low birth weight (p = 0.01, OR 6.1, 95% CI 0.8–44.4), being a male (p = 0.06, OR 9.2, 95% CI 1.3–66.9), severe thrombocytopenia (p = 0.07, OR 3.9, 95% CI 1.2–13.0), and failure to achieve microbiological clearance (p < 0.001, OR 19.6, 95% CI 4.0–98.0). The above listed factors can in combination or singly predispose individuals to *K. pneumoniae* colonization and infections.

## Discussions

*Klebsiella pneumoniae* is rapidly becoming known for its resistance properties to most of the last-line antibiotics that are usually used. It is especially problematic in hospitals, where it causes a range of acute infections. The increasing trends in the isolation rate of *K. pneumoniae* is of much concern. Economically developed areas such as China have a more advanced medical system which may increase the chance of exposure to antibiotics and this will increase the possibility of bacterial resistance. In China, the higher population density may also have increased the isolation rate among the population. In this review, although there is an increasing trend in the isolation rate, their resistance rates were not in tandem as this was evident in the decreasing trends over the years. Although, imipenem and meropenem have shown good activity against Enterobacteriaceae [22], the situation observed in this review reiterates the public health implications of *K. pneumoniae.* In Fig. 1, there is a steady increase in resistance of imipenem over the years and this can be as a result of their increasing use among the populace. Generally, the decreasing resistant rate of *K. pneumoniae* to most of the antimicrobials in China can be attributed to; (i) the enforcement of taking various actions for prevention of bacterial infection such as separating the pathogen carriers and enforcement of hand sanitization of medical professionals by the government through the Nosocomial Infection Control Committee, (ii) the restriction and control of the use of antibiotics by the Chinese Ministry of Hygiene, which has implemented guidelines for the rational use of antibiotics since 2006.

The global emergence and spread of genes of antimicrobial resistance such as ESBL and carbapenemase genes in *K. pneumoniae* isolates present a significant danger to public health. This is because carbapenems have long been deemed as the last therapeutic resort or option of antibiotics used to treat diseases and infections caused by multidrug-resistant gram-negative bacteria. The rapid global emergence of *K. pneumoniae* strains, resistant to almost all β-lactams, including carbapenems as seen in this study shows the organism’s ability to react quickly to selective environmental pressure modifications. The extensive use and misuse of carbapenems is one of the attributable reasons that has led to the evolution of plasmid-mediated carbapenemases, i.e. enzymes that hydrolyze all β-lactams including the last-line carbapenems [23]. Different resistance-mediated genes mediate antimicrobial drug resistance in *K. pneumoniae*. The high rate of resistance to carbapenems (imipenem and meropenem) observed in this study can be partly be attributed to the presence of some carbapenemase resistant-mediated genes such as bla_OXA_. bla _NDM_ and bla_KPC_ realized in this study. In *K. pneumoniae*, the bla_KPC_ genes which confers reduced susceptibility or resistance to nearly all β-lactam antibiotics by various enterobacteria are mostly carried on plasmids. The detection of carbapenemases is important from an epidemiological perspective as they are plasmid-mediated and may be transferred horizontally between different bacterial species [24]. Dissemination of resistant determinants have been recognized as a major challenge in the treatment of bacterial infections worldwide [25]. Also, resistance of *K. pneumoniae* to cephalosporin (ceftazidime, cefepime and cefotaxime) as seen in this study can also be partly be attributed to the KPC gene because the KPC enzyme hydrolyzes extended-spectrum cephalosporins. This can therefore be used to identify KPC-mediated genes that are resistant to these cephalosporins (e.g. ceftazidime, ceftriaxone, and cefotaxime).

*Klebsiella pneumoniae* resistance to Aminoglycosides (amikacin and gentamicin) as seen in this review may be as a result of modifications in cell permeability due to alterations in AcrAB-TolC and KpnEF efflux pump systems and due to loss of putative porin, KpnO. Also the disruptions in AcrAB-TolC may increase the susceptibility of *K. pneumoniae* to gentamicin [26]. The 16S rRNA methylases which are encoded on the plasmids [27] confers resistance to all aminoglycosides. Mutations which confer resistance via target modification can also be a possible attributable reason for the increasing resistance of *K. pneumoniae* to most aminoglycosides.

The low prevalence of Polymyxin (Colistin) resistance in this review makes a lot of sense because of their restricted use in human medicine dating back between the 1980s and 2000s, due to their recognized toxicity. According to Falagas and Kasiakou [28], Polymyxin works by disrupting the membrane integrity through displacement of cations (Ca^2+^/Mg^2+^) in the outer membrane, by binding to the negatively charged lipopolysaccharides (LPS) which leads to cell lysis. Undoubtedly, the presence of resistance determinants will allow *K. pneumoniae* strains to survive the barrage of antibiotics used in treatment of hospital infections.

According to a report by Dong et al. [29], *K. pneumoniae* utilize an array of virulence factors to colonize and propagate in a host cell. These include at least (a) surface antigen, particularly capsular polysaccharide (CPS, K antigen); (b) siderophores responsible for binding ferric iron secreted by the host’s iron-binding proteins; and (c) adherence variables responsible for binding to host cell surfaces, such as fimbriae type 1 and type 3, and non-fimbrial adherence proteins.

Hypermucoviscous phenotype and mucoviscosity-related genes, genes for biosynthesis of lipopolysaccharide, iron uptake and transport genes and Adhesive genes are all virulent factors that are employed by *K. pneumoniae* strains in pathogenesis. Iron is a key component for *K. pneumoniae’s* survival. As free iron is scarce in host plasma, *K. Pneumoniae* acquires iron predominantly through the secretion of siderophores; molecules with a greater iron affinity than the host transport proteins [30]. Among the siderophores secreted by *K. pneumoniae*, aerobactin is considers the most important virulent factor [31, 32], as it can cause severe infection by assisting in the transport of the organism from the intestinal tract to various tissues and also the multiplication of these organisms in the tissues.

The rmpA and rmpA2 are Plasmid-carried genes, which contribute to the enhancement of capsular production. Also, the MagA gene is an important gene used by *K. pneumoniae* strains to demonstrate an extraordinarily high resistance to human serum and phagocytosis. This gene can be used as a molecular marker for quick diagnosis and can also be useful in tracing the roots of emerging infectious diseases caused by *K. pneumoniae.* According to Fang et al. [33], the MagA protein could be a good candidate for new drug targets.

It has been shown that fimbriae contribute to diseases of the urinary tract [34], mediates the development of biofilms and also it is involve in the adherence of the organism to medical devices. Therefore, expression of these genes could improve *K. pneumoniae’s* adhesive ability to respiratory epithelial cells and also to surfaces of other medical devices such as ventilators, thereby, increasing their ability to cause ventilator-associated diseases. Fimbria, according to Huang et al. [35] and Stahlhut et al. [36], may be a major factor for biofilm-associated diseases and host entry abilities of *K. pneumoniae.*

### Limitations

Although, very positive results were obtained from this meta-analysis, it is worth noting that there were a number of limitations with this meta-analysis. This study did not utilized data from abstracts, posters and conference proceedings but only full paper publications. Because of the limited number of studies included in this meta-analysis, this study did not assess for publication bias because according to Terrin and Schmid [37] and Thornton and Lee [38], funnel plots and statistical tests for detection of publication bias are unreliable for limited number of studies.

## Conclusions

It can be concluded that antimicrobial resistance in *K. pneumoniae is* a clear and present danger in Asia which needs strong surveillance to curb this menace. Although low resistance rate to colistin and imipenem were recorded in this review, more cautious efforts should be made to develop new line of antimicrobials as resistance to these drugs are surging. It is very important for public healthcare departments to monitor and report the changes in antimicrobial-resistant isolates. This will guide doctors in prescribing the proper antimicrobials in case of resistance gene evolution. It is essential to design a preparedness plan in order to tackle this public health danger. On the other side, control measures must include a multi-faceted strategy coordinated by the domestic health officials in fields.

## Data Availability

The datasets used and/or analyzed during the current study are available from the corresponding author on reasonable request.
